# Supplementary figures and images for: Divergent Contribution of the Golgi Apparatus to Microtubule Organization in Related Cell Lines
Source: Int J Mol Sci. 2022 Dec 19;23(24):16178. doi: 10.3390/ijms232416178 (PMC9782006; doi:10.3390/ijms232416178)

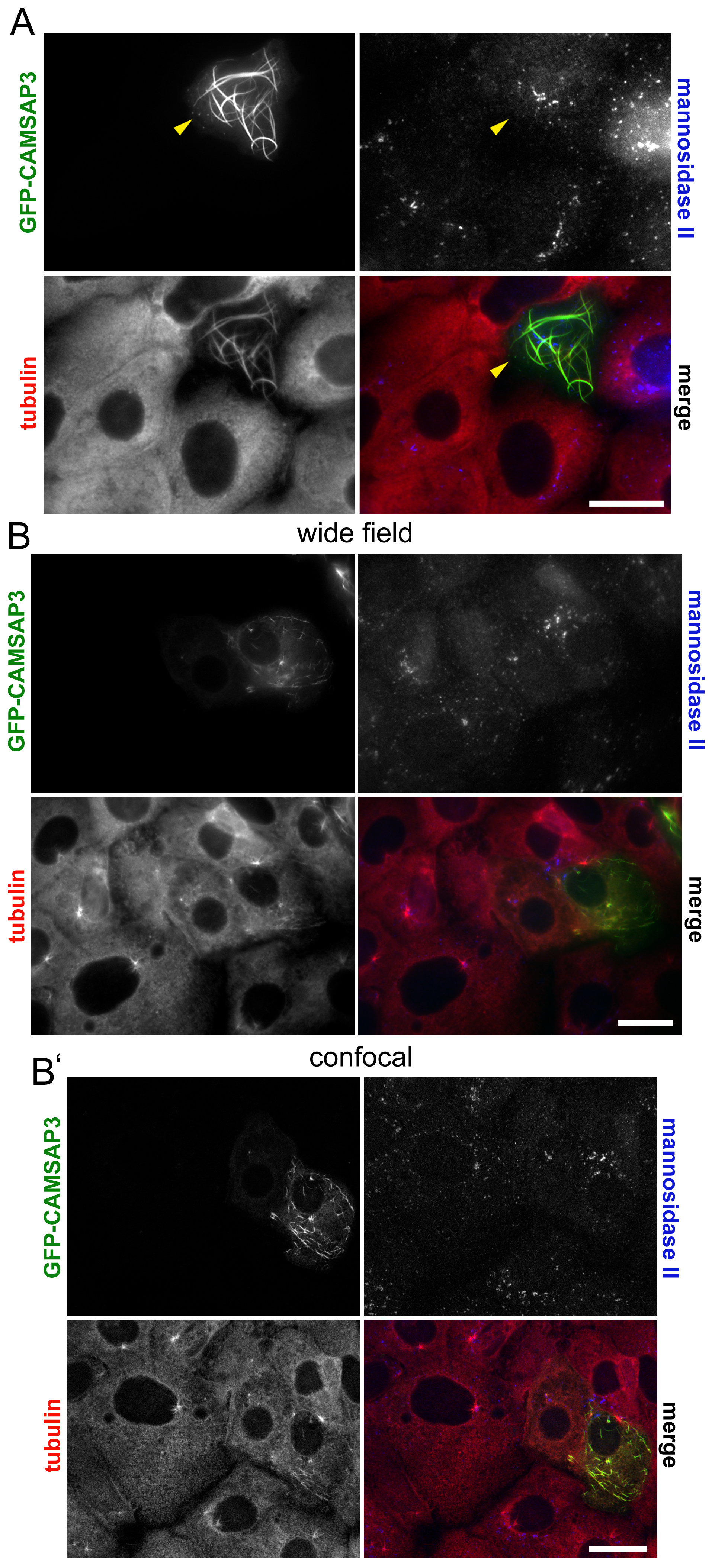

Supplement: Supplementary file 1 [file ijms-23-16178-s001.zip › Figure S3.jpg]

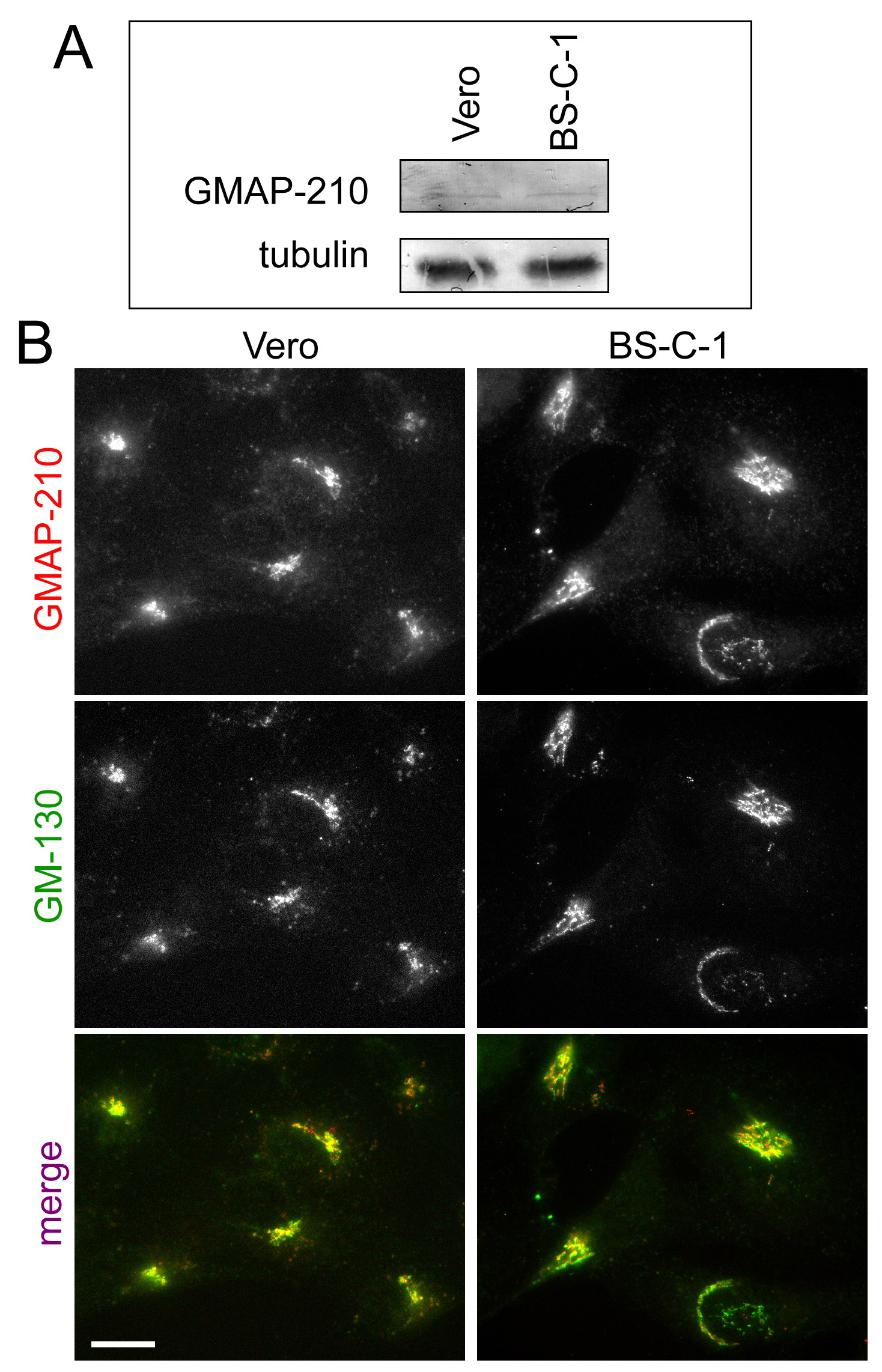

Supplement: Supplementary file 1 [file ijms-23-16178-s001.zip › Figure S4.jpg]

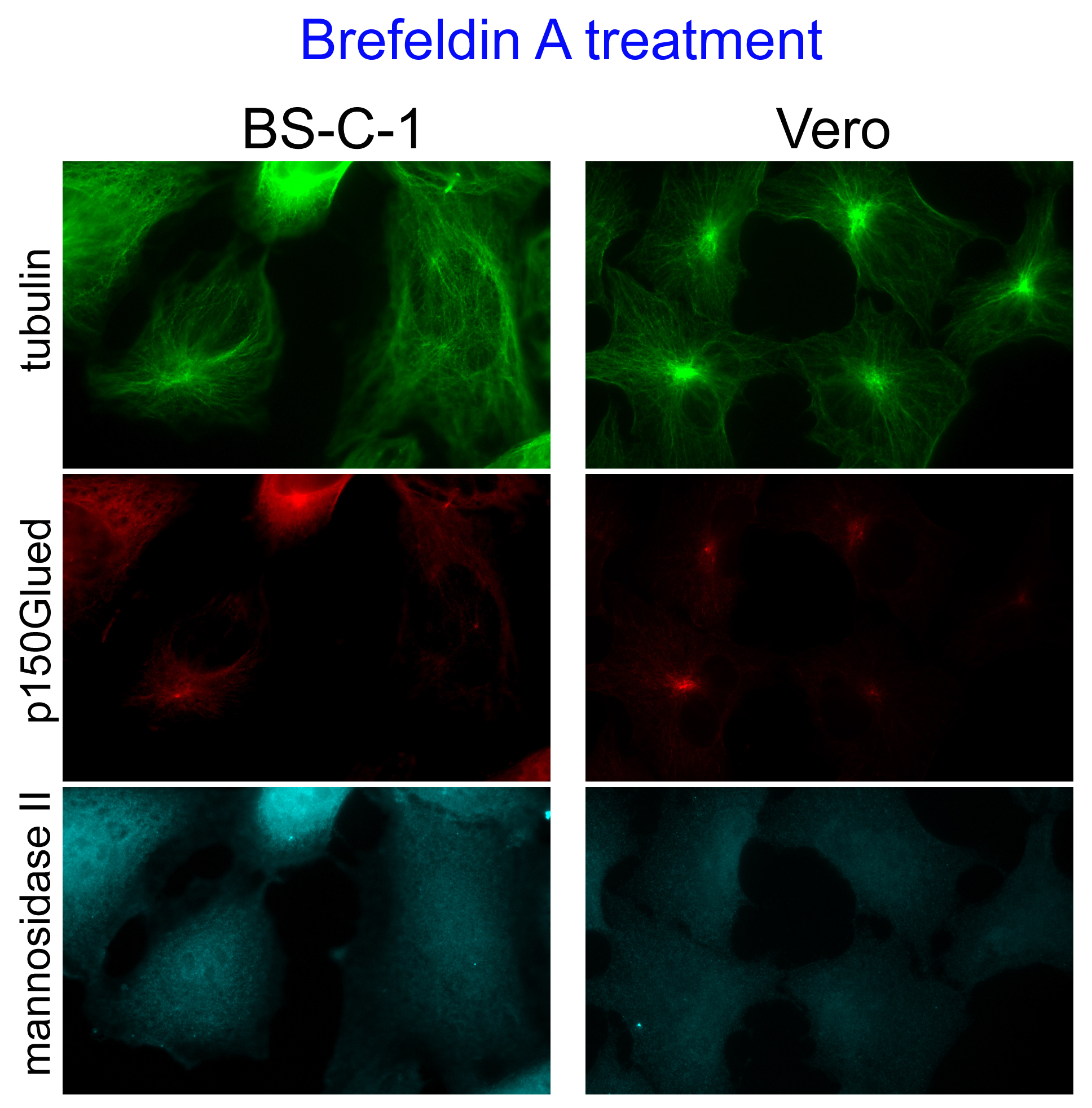

Supplement: Supplementary file 1 [file ijms-23-16178-s001.zip › Figure S1.jpg]

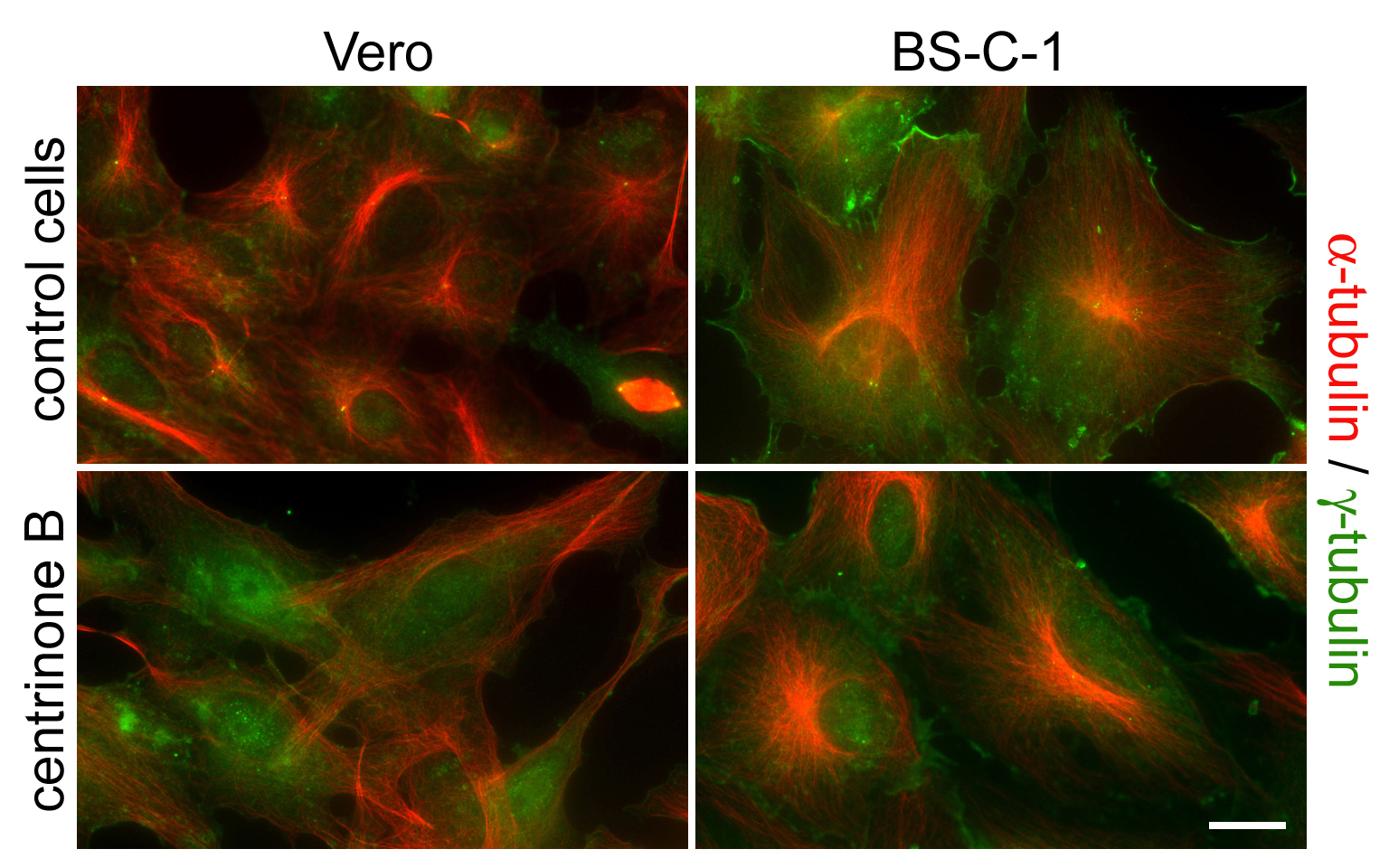

Supplement: Supplementary file 1 [file ijms-23-16178-s001.zip › Figure S2.jpg]
